# Influence of culture conditions on metabolite diversity in *Epichloë sinensis* strains

**DOI:** 10.3389/fmicb.2026.1739311

**Published:** 2026-02-24

**Authors:** Junying Liu, Wenbo Xu, Pei Tian

**Affiliations:** State Key Laboratory of Herbage Improvement and Grassland Argo-Ecosystems, Key Laboratory of Grassland Livestock Industry Innovation, Ministry of Agriculture and Rural Affairs, Engineering Research Center of Grassland Industry, Ministry of Education, College of Pastoral Agriculture Science and Technology, Lanzhou University, Lanzhou, China

**Keywords:** GC-MS, hyphae, *in vitro*, LC-MS, PDA, PDB

## Abstract

**Introduction:**

*Epichloë sinensis*, associated with *Festuca sinensis*, exhibits rich morphological, genetic and functional diversity across different strains.

**Methods:**

To comprehensively evaluate the categories and difference in the secondary metabolites of different *E. sinensis* strains and thereby reveal the functional diversity, the present study analyzed the metabolites of mycelia collected from potato dextrose agar (PDA) and potato dextrose broth (PDB) cultures and fermentation broths of three different *E. sinensis* strains. These strains (ID1, 2, and 84C) were isolated from *F. sinensis* seeds collected from different locations.

**Results:**

These three strains of *E. sinensis* produced a total of 1029 compounds categorized into 14 major classes in the mycelia and fermentation which included a wide range of compound structure and types, revealing *E. sinensis* possesses abundant metabolites. The different strains shared the similar types of metabolites which only varied in concentrations of some metabolites, indicating the similarity in metabolic function. However, types, quantity and concentrations of secondary metabolites varied in different conditions, suggesting the metabolites changed with environmental variations, as 135 unique compounds were only detected in mycelia from PDA (solid medium), while 205 exclusive metabolites were identified in PDB fermentation broth (liquid environment). Comparing metabolites of hyphae in PDA and PDB, there were a total of 100 different compounds. Comparing metabolites of hyphae in PDB and fermentation broth, there were 136 different compounds.

**Discussion:**

These metabolites were mainly involved in the citrate cycle (TCA cycle) and alanine, aspartate, and glutamate metabolism.

## 1 Introduction

*Epichloë sinensis* can enhance plant growth, and also improve the stress resistance of host grasses—*Festuca sinensis* (Luo and Tian, 2021; Wang M. N. et al., 2021; Wang Y. B. et al., 2021; Xu et al., 2021). These improved performances were related to secondary metabolites produced by the fungal endophytes especially alkaloids, i.e., indole diterpenes, peramine, ergot and lolines (Schardl et al., 2004).

Endophytes may also produce alkaloids and some other metabolites *in vitro*, for example, under fermentation conditions, *E. inebrians* can produce indole diterpenoid alkaloids *in vitro*, *E. festucae* can produce indole diterpenes, sesquiterpenes, diacetamide, siderophores and related peptides, and *E. bromicola* can produce peptides, dipeptides, benzene derivatives, sesquiterpenes, sugars (Yue, 2000; Koulman et al., 2012; Song and Nan, 2015). The types and concentrations of these metabolites varied with conditions. For example, *E. inebrians* produced alkaloids only on SM and M104 media, and the concentration in M104 medium was higher than that in SM medium (Gao and Nan, 2007). Eight strains of *Epichloë* endophytes were cultivated on M104 medium, including two strains isolated from *A. inebrians*, two strains from *F. sinensis*, and four strains from *Elymus dahuricus* and *Hordeum vulgare*. These strains produced ergometrine, ergotamine, tremor and polyamine alkaloids, with varying levels of alkaloid content among the strains (Liu, 2019).

In addition to these alkaloids, *Epichloë* endophytes also produced other metabolites which exhibited rich functional diversity. The fermentation broth of the strains exhibited significant inhibitory effects on the mycelial growth and conidial germination of several pathogenic fungi (such as *Alternaria tenuis*, *Drechslera avenae*, *Bipolaris sorokiniana*, *Bipolaris zeae*, and *Fusarium acuminatum*) on potato dextrose agar (PDA) medium (Liu, 2019). Metabolite cyclosporin T isolated from *E. bromicola* hypha showed significant inhibitory activity against four plant pathogenic fungi—*Helminthosporium graminearum*, *Curvularia lunata*, *Fusarium avenaceum* and *Alternaria tenuis* (Song and Nan, 2015). Furthermore, the extract from the fermentation broth showed both gastric and contact toxicity against *Rhopalosiphum padi* (Liu, 2019). The compound 3′-dihydroxy-5,5′-dimethyldiphenyl ether showed significant phytotoxic effects on the roots and seedlings of *Lolium perenne* and *Poa crymophila*, respectively (Song and Nan, 2015). Enzyme linked immunosorbent assay (ELISA) analysis showed that low levels of growth hormones, i.e., indole-3-acetic acid (IAA), cytokinin (CTK), gibberellin (GA) and abscisic acid (ABA), were present in both *E. sinensis* mycelia and its culture filtrates under liquid culture conditions (Luo and Tian, 2021). These studies suggested that fungal endophytes can produce diverse metabolites with rich function.

The *Epichloë* endophytes including different *E. sinensis* strains showed great morphological and genetic diversity. For example, 3 *E. sinensis* strains which were isolated from *F. sinensis* in Xiahe, Gansu Province, Yushu, Qinghai Province and Ping’an, Qinghai Province, respectively, performed different growth rate and genetic diversity (Xu et al., 2021). Comparative analysis of the conidial, conidiophore, and colony characteristics of 48 fungal endophytes strains isolated from *F. sinensis* seeds collected from Ganjia and Sangke in Gannan, Gansu Province revealed a considerable morphological diversity among these strains (Yang et al., 2011). Further research is necessary to determine the potential impact of these differences in strains/regions and their growth environment on secondary metabolites.

The secondary metabolites produced by *E. sinensis* have exhibited antibacterial activity or insect resistance. It was found that after soaking the seeds of *F. sinensis* and *A. inebrians* in the fermentation broth of *E. sinensis*, the seed germination and seedling growth were either promoted or inhibited depending on the concentration of the fermentation broth (Zhong et al., 2017). High fermentation filtrate from *E. sinensis* significantly improved seed germination, seedling development, and radicle elongation of *L. perenne*, but reduced pathogen development (Liu et al., 2018). Eleven compounds such as sorbitol, methyl-tert butyl ether and dihydromannitol, were identified in the fermentation broth of *E. sinensis* (Zhou et al., 2019). However, the types and function of these metabolites were still unidentified.

The objectives of the present study was to investigate the functional diversity of compounds within *E. sinensis*, to examine the differential changes in these compounds due to different strains and cultivation conditions, and to establish a foundation for the development and utilization of fungal endophytes and their metabolites.

## 2 Materials and methods

### 2.1 Experimental materials

Three *E. sinensis* strains, 1, 2 and 84C, were isolated from *F. sinensis* seeds collected from Xiahe, Gansu Province (102°31′30″N, 35°12′0″E, strain 1), Qinghai Yushu (97°10′15.4″N, 32°49′38″E, strain 2), and Qinghai Ping’an (102°05.116N, 36°17.723E, strain 84C), respectively. The corresponding biological replicates for each group were 4 cases. The strains were stored at 4 °C in the Microbial Germplasm Resource Bank of the Center for Grassland Microbiome of Lanzhou University.

*E. sinensis* was grown on a sterilized PDA medium at 25 °C for 4 weeks to produce hypha. A 6-mm diameter puncher was then taken at the edge of the colony and inoculated into potato dextrose broth (PDB) and PDA media. Liquid culture was carried out using 250 mL conical flasks, containing 100 mL each of PDB medium. The three *E. sinensis* strains were inoculated and replicated 20 times per strain. The *E. sinensis* strains were shaken on an intelligent shaker at 25 °C and 145 rpm.

### 2.2 Collection of fungal endophytes hyphae and fermentation broth

Four samples were randomly collected from fungal endophytes mycelia cultured in PDB medium, PDB fermentation broth, and fungal endophytes mycelia cultured in PDA medium (Liu and Su, 2004). The fungal endophytes hyphae and fermentation broth was collected as described by Luo and Tian (2021). These samples were preserved on dry ice and subsequently transported to Biotree Biomedical Technology (Biotree Biomedical Technology Co., Ltd., Shanghai, China) for liquid chromatography-mass spectrometry (LC-MS) and gas chromatography-mass spectrometry (GC-MS) analysis.

### 2.3 Gas chromatography-mass spectrometry analysis

A steel ball was added to EP tube with a 500 mg sample. Each sample was treated for 30 s with a pre-cooled solvent (1,000 μL; methanol: acetonitrile: water = 2:2:1, including internal standard ribitol) and vortex mixed. Samples were then subjected to low-temperature ultrasound for 5 min, followed by an hour rest at −40 °C. After centrifugation at 12,000 g at 4 °C for 15 min, the supernatant was vacuum-dried (Fan et al., 2024).

The GC-MS analysis was performed as described by Bini et al. (2025) with a slight improvement. An Agilent 7890 gas chromatograph was used in conjunction with a time-of-flight mass spectrometer (PEGASUS HT, LECO). This system employed a DB-5MS capillary column (30 m × 250 μm × 0.25 μm, J&W Scientific, Folsom, CA, United States). An injection volume of 1 μL was employed. The injector temperature was set at 250 °C and operated in splitless mode. The oven temperature was programmed from 50 °C (isothermal for 1 min) to 310 °C (isothermal for 8 min) at the rate of 10 °C/min. Mass transfer line temperature was set at 280 °C. All mass spectra were acquired with an electron ionization system (EI, Electron Impact mode) with ionization energy of 70 eV and source temperature of 250 °C.

The Chromatof program (v4.3x, LECO) was used to analyse the mass spectrometry data, encompassing peak extraction, baseline correction, deconvolution, peak integration, and peak alignment. The LECO Fiehn RTX5 database was utilised for the qualitative analysis of the substances. Removed ion peaks with detection rates below 50% or a relative standard deviation (RSD) exceeding 30% in samples.

### 2.4 Liquid chromatography-mass spectrometry analysis

A vacuum freeze-drier (Scientz-100F) was used to dry the samples, which were then ground with a mixer mill (MM400, Retsch) and zirconia bead for 4 min at 35 Hz until it became a fine powder. Lyophilized powder (50 mg) was dissolved with 1 mL of extract solution (methanol: acetonitrile: water = 2: 2: 1) (3× total). The samples were incubated for 1 h at −40 °C, centrifuged at 4 °C and 12,000 rpm for 15 min, and filtrated prior to UPLC-MS/MS analysis (Zhang et al., 2023). Quality control (QC) samples were then prepared by combining all of the sample extracts.

The LC-MS analysis was performed as described by Jiang et al. (2023). A Vanquish UHPLC system (ThermoFisher, Germany) was used in conjunction with a UPLC BEH Amide column (2.1 mm × 100 mm, 1.7 μm) linked to a Q Exactive HFX mass spectrometer (Orbitrap MS, Thermo) to separate the target compound. The mobile phase included 25 mmol/L ammonium acetate and 25 mmol/L ammonia hydroxide in water (pH = 9.75) (A) and acetonitrile (B). The auto-sampler temperature was 4 °C, and the injection volume was 3 μL. The QE HFX mass spectrometer was utilised for its capability to obtain MS/MS spectra in information-dependent acquisition (IDA) mode, governed by the acquisition software (Xcalibur, Thermo). In this mode, the acquisition program perpetually assesses the complete scan MS spectrum. The Q Exactive TMHF-X mass spectrometer was operated in positive/negative polarity mode with a spray voltage of 3.2 kV, a capillary temperature of 350 °C, a sheath gas flow rate of 30 arb, and an auxiliary gas flow rate of 25 arb.

The raw data were first converted to mzXML format by ProteoWizard software package (v3.0.8789) (Wu and Li, 2012). Subsequently, R XCMS software package was used for feature detection, retention time correction, and alignment (Merixell et al., 2015). Finally, these were aligned with the biotreedb (v2.1) self-constructed secondary mass spectrometry database for substance annotation. The algorithm score threshold was set at 0.3.

### 2.5 Data analysis

A series of preparatory procedures and collation of mass spectrometry data were carried out (Dunn, 2011). The relative content of each component was calculated using the method of peak area normalization. The relative content of the top approximately 100 compounds in each of the strains was assessed from GC-MS and LC-MS by one-way analysis of variance (ANOVA) using SPSS19.0 (IBM Corporation, Armonk, NY, United States), with multiple comparisons using the Duncan method. The relative concentrations of common metabolites were calculated by the following formulas.


$$\mathrm{RT}(\%)=\frac{\text{TAIC}}{\mathrm{TCA}}\times 100\%$$

Where RT are the relative content, TAIC are the total area of identified compounds, TCA are the total chromatogram area.

The metabolite data were log_2_-transformed (log2) to improve normality and mean centering before OPLS-DA analysis by SIMCA-16.0 software (Umetricus AB, Sweden). The variable importance in the projection (VIP) ≥1 in the OPLS-DA model and the absolute Log_2_FC (fold change) ≥1 were set for screening differential metabolites. Those having a *p*-value of *t*-test 0.05 and VIP ≥1 were deemed to be different between the two groups. Venn diagrams were employed to illustrate the number of divergent metabolites. An enrichment analysis of differential metabolites was conducted using the KEGG database.^1^ KEGG pathway enrichment analysis was performed using the Metware Cloud, a free online platform for data analysis.^2^

## 3 Results

### 3.1 Main types and quantities of metabolites produced by the fungal endophytes strains from the combination of LC and GC-MS analysis

**Figure 1:**
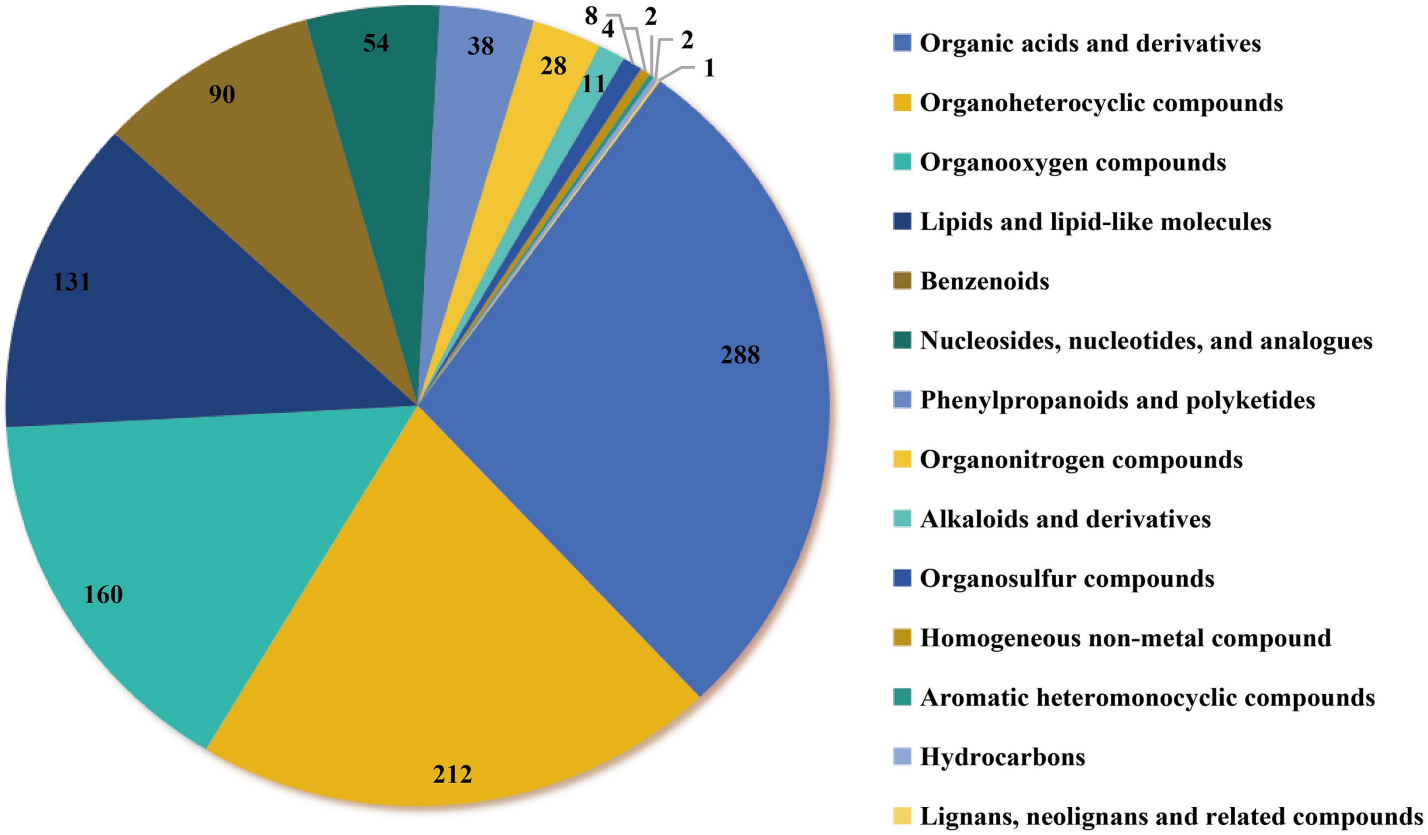
The metabolites class distribution of *E. sinensis* based on LC and GC-MS analysis.

The fungal endophytes produced a total of 1,029 secondary metabolites categorized into 14 major classes under *in vitro* culture conditions (Figure 1). The metabolites consisted of organic acids and derivatives (288 in total, including 40 amino acids and isomers), organoheterocyclic compounds (212), organooxygenates (160), lipids and lipid-like molecules (131), benzeneoids (90), nucleosides, nucleotides, and analogues (54), phenylpropanoids and polyketides (38), organonitrogen compounds (28), alkaloids and derivatives (11), organosulfur compounds (8), homogeneous non-metal compounds (4), aromatic monoheterocyclic compounds (2), hydrocarbons (2), and lignans, neolignans, and related metabolites (1).

### 3.2 Differential metabolites of three strains from the combination of LC and GC-MS analysis

**Figure 2:**
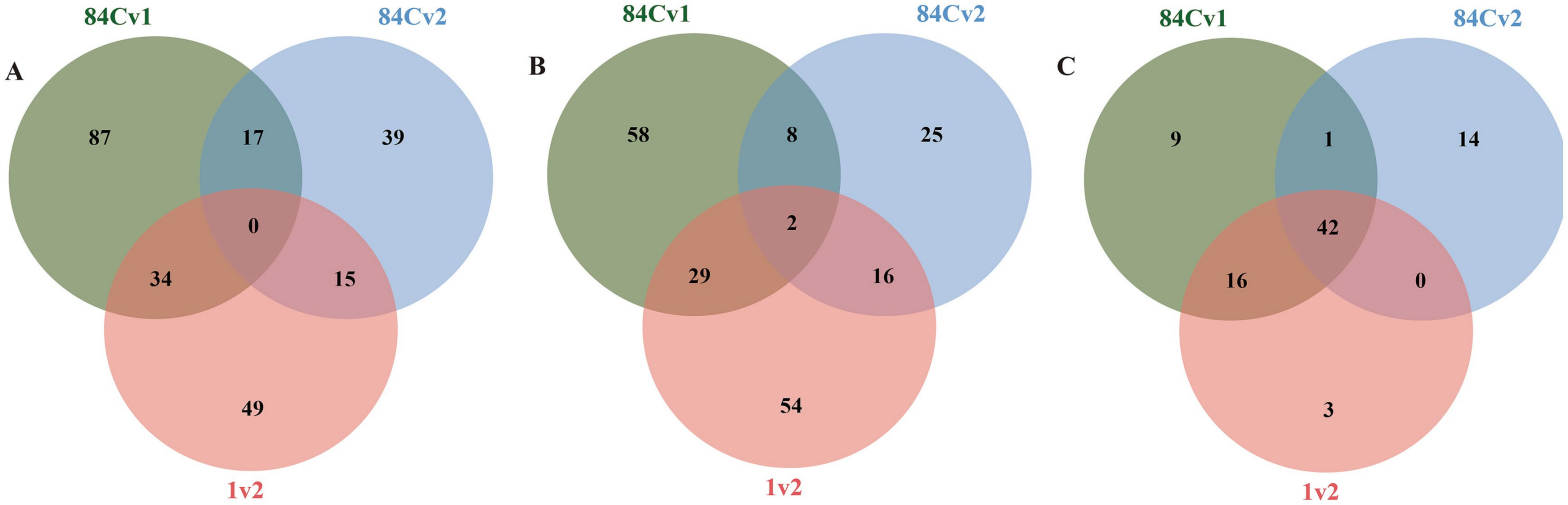
The presence of divergent metabolites observed among strains cultivated under different conditions. **(A)** Differential metabolites among strains in PDB mycelium. **(B)** Differential metabolites among strains in PDB broth. **(C)** Differential metabolites among strains in PDA.

The types of compounds produced by three *E. sinensis* strains under different conditions varied (Figure 2).

In the PDB harvested mycelium, there were 98 differential metabolites between strains 1 and 2, 138 differential metabolites between strains 1 and 84C, and 71 differential metabolites between strains 2 and 84C (Figure 2A). There were no common differential metabolites among the three strains.

In PDB fermentation broth, there were 101 differential metabolites between strains 1 and 2, 97 between strains 1 and 84C, and 51 between strains 2 and 84C (Figure 2B). Only two compounds, 2-cyclohexanol B epoxide 2 and methyl n-acetylanthranilate, were common across all three strains.

In PDA harvested mycelia, there were 61 differential metabolites between strains 1 and 2, 68 between strains 1 and 84C, and 57 between strains 2 and 84C (Figure 2C). Among them, 42 compounds were common in all three strains.

**Figure 3:**
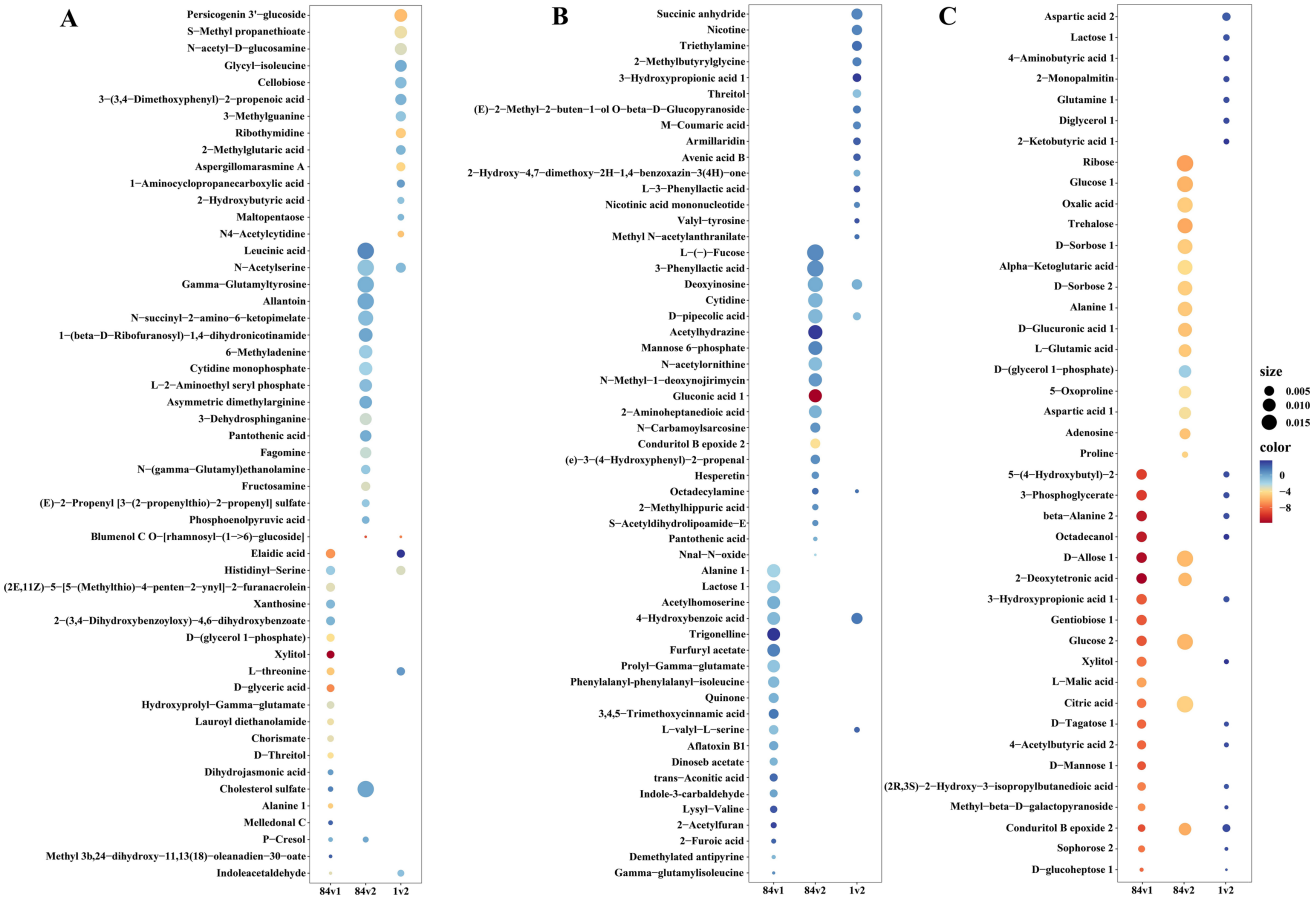
The top 20 differential metabolites of the significance of the up-regulated and down-regulated differential metabolites on bubble diagram. **(A)** Differential metabolites among strains in PDB mycelium. **(B)** Differential metabolites among strains in PDB broth. **(C)** Differential metabolites among strains in PDA. The *X*-axis represents the rich factor and the *Y*-axis represents the name of the path. The size of the bubbles indicates the number of differential metabolites involved, and the color of the bubbles indicates the degree of enrichment of the pathway.

The bubble diagram shows the top 20 up- and down-regulated differential metabolites (VIP ≥ 1, *p* < 0.05) under different culture conditions (Figure 3). In PDB mycelium, differential metabolites that altered between two strains (84C vs. 1, 84C vs. 2, 1 vs. 2) did not overlap (Figure 3A). For example, upregulated metabolites (e.g., methyl 3b, p-cresol) and downregulated compounds (e.g., indoleacetaldehyde) were found only for 84C vs. 1 comparison. For 84C vs. 2 comparison, the downregulated metabolite, blumenol C co-[rhamnosyl-6-O-glucoside], was unique to this group.

In PDB broth, differential metabolites (e.g., gamma glutamylisoleucine, aflatoxin B1 in 84C vs. 1; s-acetyldihydrolipoamide-e in 84C vs. 2) did not overlap either over groups (Figure 3B).

In mycelia harvested from PDA, Conduritol b epoxide was a commonly shared differential metabolites all strains (84C vs. 1, 84C vs. 2, 1 vs. 2) (Figure 3C). Compounds such as D-glucoheptose 1, sophorose 2, and 3-hydroxypropionic acid 1 were repeatedly observed in multiple comparisons (e.g., 84C vs. 1 and 1 vs. 2).

### 3.3 Effects of culture conditions on the type and quantity of metabolites from the combination of LC and GC-MS analysis

**Figure 4:**
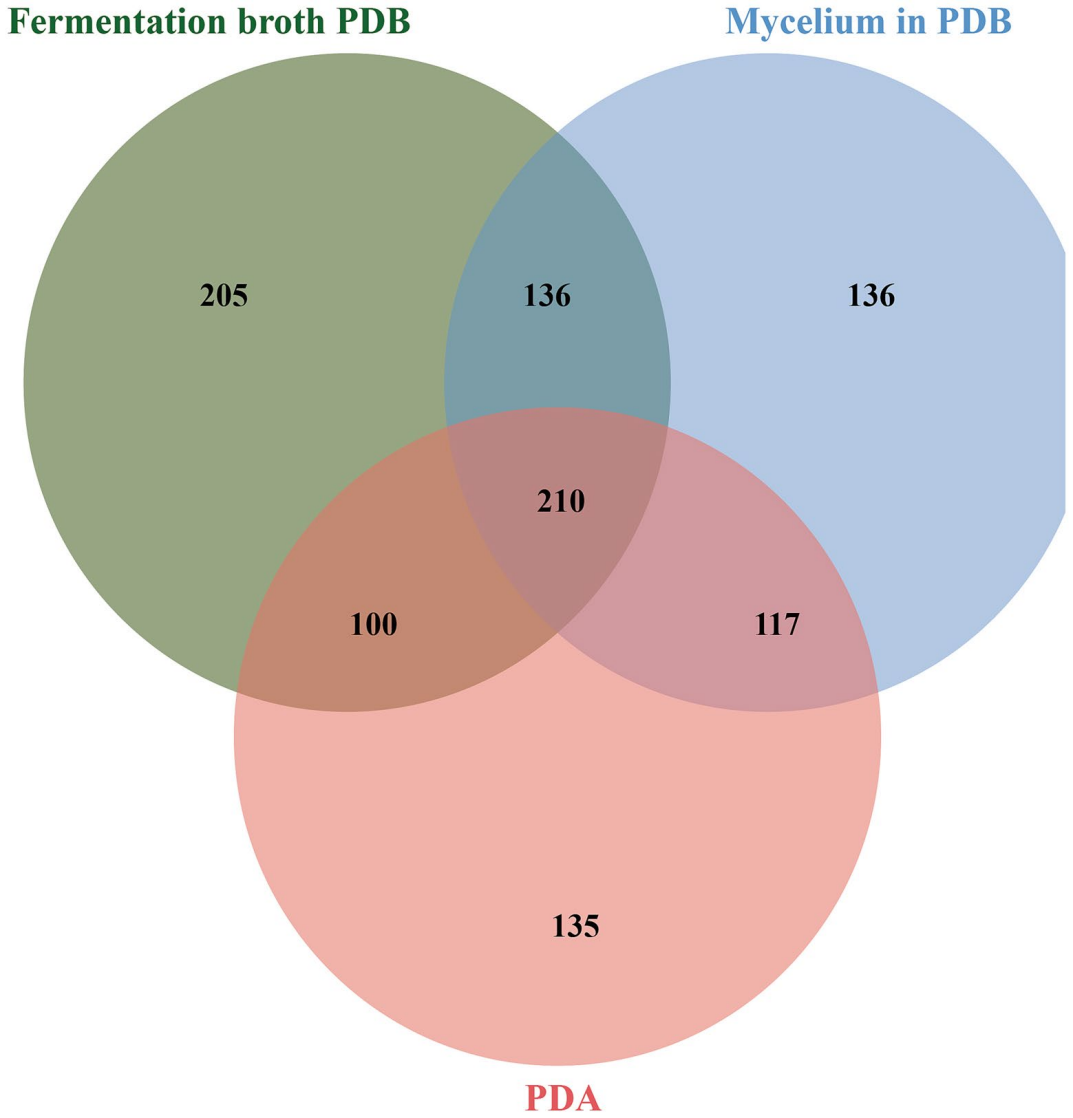
Quantitative difference of tested compounds under different culture conditions.

There were differences in the type and quantity of metabolites produced by a same strain under different culture conditions (PDA harvested mycelium, PDB harvested mycelium and fermentation broth) (Figure 4). In the three strains, 1,029 compounds were detected with 210 commonly shared metabolites (both positive and negative ions) across all culture conditions. These included malic acid, mannose, sorbitol, succinic acid, threitol, and trehalose. There were a total of 117 commonly shared metabolites (both positive and negative ions) present within the mycelia of the three strains (including PDA and PDB), but not in the fermentation broth of the strains. There were 100 compounds in the fermentation broth and PDA-harvested mycelia, but not in the PDB-harvested mycelia. There were 136 compounds in the PDB-harvested mycelia and fermentation broth, but not in the PDA-harvested mycelia. There were 135 distinct metabolites in the PDA-harvested mycelia of the three strains. However, these metabolites were not detected in the mycelia or fermentation broth cultivated with PDB for the three strains. A total of 205 metabolites were detected in the fermentation broth of all three strains, but not in the mycelia of the strains (both under PDA and PDB). A total of 136 metabolites were identified in the mycelia obtained from PDB, but not in the mycelia and fermentation broth under PDA.

### 3.4 Metabolic pathways of different metabolites among the tested strains under different culture conditions

**Figure 5:**
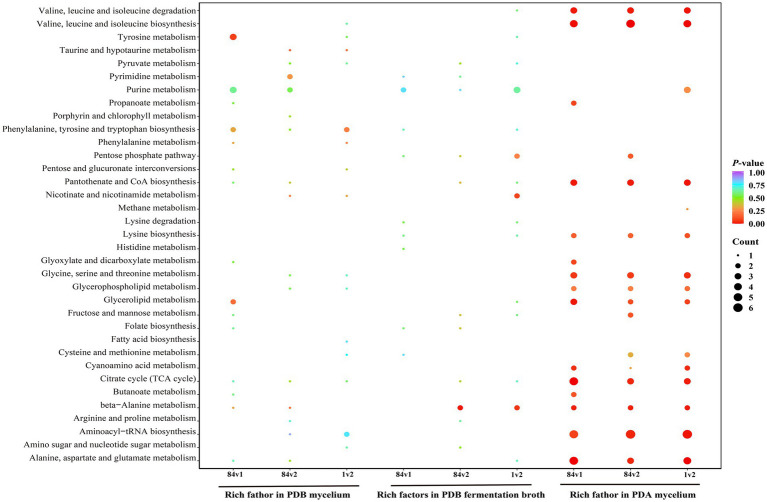
KEGG pathway enrichment analysis of the differential metabolites under different culture conditions. The *X*-axis represents the rich factor and the *Y*-axis represents the name of the path. The size of the bubbles indicates the number of differential metabolites involved, and the color of the bubbles indicates the degree of enrichment of the pathway.

The combination of LC-MS and GC-MS analysis revealed a comparison in PDB mycelium between strains 84C and 1, demonstrating enrichment in 15 metabolic pathways, including tyrosine metabolism, glycerolipid metabolism, phenylalanine metabolism, and beta-alanine metabolism (Figure 5). Comparison of strains 84C and 2 found enrichment in 15 pathways such as taurine and hypotaurine metabolism, nicotinate and nicotinamide metabolism, and porphyrin and chlorophyll metabolism. In the comparison of strains 1 and 2, an overlap of 15 enriched pathways was observed, encompassing several shared pathways (the citrate cycle (TCA cycle) and alanine, aspartate, and glutamate metabolism) as well as novel pathways (valine, leucine, and isoleucine biosynthesis).

The differential compounds in PDB broth between strains 84C and 1 were enriched in fewer pathways (9) such as histidine metabolism and lysine degradation (Figure 5). In contrast, the comparison between strains 84C and 2 involved 11 pathways such as beta-alanine metabolism and pantothenate and CoA biosynthesis.

The comparisons in PDA-harvested mycelia between strains 84C vs. 1, 84C vs. 2, and 1 vs. 2 revealed 15 metabolic pathways: amino acid metabolism such as alanine, aspartate, and glutamate, fatty acid-related pathways such as glycerolipid metabolism, and energy production cycles such as the citrate cycle (Figure 5). However, each pair-wise comparison also had its unique pathways, e.g., cyanoamino acid metabolism in the strain 1 vs. 2 comparison.

### 3.5 Relative content of commonly shared metabolites among the tested strains under different culture conditions

The relative concentrations of six commonly shared metabolites generated by the three strains across three culture conditions were assessed using GC-MS detection methods (Table 1). The relative levels of commonly shared metabolites exhibited significant variations both within the same strain across different culture conditions and among different strains under the same culture condition. The relative content of malic acid produced by strain 1 in PDB harvested mycelia was significantly higher from that in fermentation broth and PDA mycelia (*p* < 0.05). The relative content of malic acid produced by strains 2 and 84C in PDA harvested mycelia was significantly greater than that in PDB harvested mycelia and fermentation broth (*p* < 0.05). In PDB-cultivated mycelia, the relative content of malic acid produced by strain 1 was significantly higher than that by strain 2 (*p* < 0.05). The relative content of malic acid produced by strain 84C in PDB fermentation broth and PDA harvested mycelia was significantly higher than that by strains 1 and 2 (*p* < 0.05). The sorbitol content was significantly greater in PDA-harvested mycelia than in PDB-harvested mycelia (*p* < 0.05), and in PDB-harvested mycelia than in the fermentation broth (*p* < 0.05). In PDB-cultivated mycelia, the sorbitol content produced by strain 84C was significantly higher than that by strains 1 and 2 (*p* < 0.05). For the fermentation broth, the relative content of sorbitol produced by strain 84C was significantly lower than by strains 1 and 2 (*p* < 0.05). In PDA-harvested mycelia, the relative content of sorbitol produced by strain 1 was significantly higher than that by strains 2 and 84C (*p* < 0.05).

**Table 1:** Analysis of the relative content of commonly shared metabolites in three *E. sinensis* strains based on gas chromatography-mass spectrometry (GC-MS).

Metabolites	Relative content of mycelium in PDB (%)			Relative content of fermentation broth in PDB (%)			Relative content of mycelium in PDA (%)		
	1	2	84C	1	2	84C	1	2	84C
L-Malic acid	8.54 ± 0.89aA	4.67 ± 0.45bB	6.64 ± 0.87abB	1.17 ± 0.13bC	2.09 ± 0.28bC	3.97 ± 0.59aC	4.76 ± 0.53cB	10.06 ± 0.96bA	18.05 ± 2.34aA
Mannose	0.36 ± 0.09aA	0.22 ± 0.03abA	0.10 ± 0.02bB	0.01 ± 0.001cC	0.10 ± 0.01bB	0.19 ± 0.05aA	0.14 ± 0.03aB	0.09 ± 0.01bB	0.10 ± 0.01ab
Sorbitol	0.61 ± 0.06bB	0.49 ± 0.08bB	0.89 ± 0.12aB	0.22 ± 0.02aC	0.25 ± 0.07aC	0.15 ± 0.04bC	2.64 ± 0.23aA	1.94 ± 0.30bA	1.90 ± 0.04bA
Succinic acid	0.50 ± 0.05aA	0.39 ± 0.05abB	0.30 ± 0.04bB	0.16 ± 0.02aC	0.17 ± 0.01aC	0.18 ± 0.06aB	0.37 ± 0.02cB	0.73 ± 0.09bA	0.89 ± 0.04aA
Threitol	0.54 ± 0.05aA	0.35 ± 0.09bA	0.32 ± 0.02bA	0.10 ± 0.01aB	0.20 ± 0.01aB	0.12 ± 0.06aB	0.04 ± 0.001bC	0.03 ± 0.001bB	0.09 ± 0.02aB
Trehalose	0.44 ± 0.10aB	0.45 ± 0.13aB	0.23 ± 0.06aB	0.04 ± 0.001bC	0.07 ± 0.01aC	0.05 ± 0.01abC	33.36 ± 5.62abA	36.43 ± 2.85aA	22.55 ± 2.01bA

Values are the means ± standard errors. Different lower-case letters indicate significant difference between strains under the same culture condition at *p* < 0.05. Different upper-case letters indicate significant differences in the same strain under different culture conditions at *p* < 0.05.

Among all metabolites detected by LC-MS, the three selected strains were screened for commonly shared metabolites among the top 100 compounds with the highest relative content under different culture conditions, and a comparison of the relative content of metabolites was conducted (Table 2). Sixteen compounds were screened for positive ions, and 20 commonly shared metabolites were screened for negative ions (Table 3). The distribution of these compounds was analogous to that identified by GC-MS. Significant differences were detected in the relative content of metabolites within the same strain under varying culture conditions, as well as among different strains under identical culture conditions. Using adenylate content as an example, the relative adenylate levels produced by strains 1 and 2 in PDB and PDA harvested mycelia were significantly higher than that in fermentation broth (*p* < 0.05). Conversely, strain 84C had a significantly greater relative adenylate content in fermentation broth than in PDB and PDA harvested mycelia (*p* < 0.05) (Table 2). In PDB harvested mycelia, strain 2 had a significantly higher relative content of adenylate than strain 84C (*p* < 0.05). In the PDB broth and PDA harvested mycelia, no significant differences were observed in the relative content of adenylate among the three strains (Table 2). The relative content of proline produced by the three strains in PBD mycelia was significantly higher than that in PDB fermentation broth and PDA harvested mycelia (*p* < 0.05). However, the proline content produced by the three strains across the three culture conditions had no significant differences (Table 2). The relative content of sorbitol in mycelia harvested PDB was significantly higher than that in mycelia harvested PDA (*p* < 0.05), and the relative content of sorbitol in mycelia harvested by PDA was significantly greater than that in the fermentation broth (*p* < 0.05). In PDB-cultivated mycelia, the relative content of oleic acid produced by strain 1 was significantly higher than that by strain 2 (*p* < 0.05). The fermentation broth had no significant variation in the relative content of oleic acid produced by the three strains (*p* > 0.05). In PDA harvested mycelia, the relative oleic acid content of strain 1 was significantly higher than that of strains 2 and 84C (*p* < 0.05; Table 3). The inositol content produced by the three strains in fermentation broth was significantly higher than that in PDB and PDA (*p* < 0.05). The relative content of mycelia in PDB was significantly greater than that in mycelia in PDA (*p* < 0.05). The variation in inositol content among the three strains across all three culture conditions was not statistically significant (Table 3).

**Table 2:** Analysis of the relative content of commonly shared metabolites among three *E. sinensis* strains based on liquid chromatograph-mass spectrometer (LC-MS) positive ion (POS).

Metabolites	Relative content of mycelium in PDB (%)			Relative content of fermentation broth in PDB (%)			Relative content of mycelium in PDA (%)		
	1	2	84C	1	2	84C	1	2	84C
Adenosine	4.61 ± 1.06abA	5.40 ± 1.27aA	3.29 ± 0.59bB	0.94 ± 0.19aB	0.64 ± 0.04bB	0.98 ± 0.25aC	4.25 ± 0.87aA	4.99 ± 0.52aA	4.52 ± 0.34aA
D-Proline	1.34 ± 0.33aA	1.46 ± 0.10aA	0.98 ± 0.28aA	0.33 ± 0.08aB	0.23 ± 0.08aB	0.28 ± 0.12aB	0.02 ± 0.01aB	0.03 ± 0.01aC	0.03 ± 0.01aB
L-Acetylcarnitine	1.30 ± 0.45aA	1.02 ± 0.15aB	1.18 ± 0.18aA	0.45 ± 0.10aA	0.20 ± 0.07bC	0.33 ± 0.12abB	1.14 ± 0.07bB	1.31 ± 0.06aA	1.04 ± 0.09bA
L-Carnitine	0.64 ± 0.10aB	0.76 ± 0.15aB	0.78 ± 0.17aB	0.46 ± 0.04bC	0.25 ± 0.06cC	0.81 ± 0.12aB	1.52 ± 0.09aA	1.34 ± 0.03bA	1.36 ± 0.08bA
L-Tyrosine	0.63 ± 0.13aA	0.62 ± 0.12aA	0.55 ± 0.13aA	0.16 ± 0.03aC	0.09 ± 0.03aC	0.14 ± 0.08aB	0.43 ± 0.01aB	0.44 ± 0.01aB	0.43 ± 0.01aA
Betaine	0.48 ± 0.13aB	0.34 ± 0.05aB	0.40 ± 0.07aB	0.30 ± 0.04aB	0.24 ± 0.03bB	0.16 ± 0.02cC	2.48 ± 0.53aA	2.27 ± 0.11aA	2.79 ± 0.18aA
Choline	0.19 ± 0.04bA	0.23 ± 0.02bA	0.41 ± 0.15aA	0.19 ± 0.07abA	0.10 ± 0.04bB	0.25 ± 0.07aA	0.26 ± 0.16aA	0.14 ± 0.01aB	0.19 ± 0.171aA
Butyrylcarnitine	0.13 ± 0.04bB	0.12 ± 0.04bB	0.27 ± 0.05aB	0.06 ± 0.02bC	0.03 ± 0.02bC	0.20 ± 0.07aB	0.24 ± 0.03bA	0.18 ± 0.02bA	0.43 ± 0.09aA
Pyridoxine	0.11 ± 0.03aA	0.11 ± 0.04aA	0.09 ± 0.03aA	0.62 ± 0.06aB	0.78 ± 0.09aB	0.58 ± 0.16aB	0.02 ± 0.01bC	0.03 ± 0.01aB	0.01 ± 0.01cB
3-Dehydroxycarnitine	0.11 ± 0.01aB	0.16 ± 0.09aC	0.09 ± 0.05aB	0.03 ± 0.01aB	0.04 ± 0.01aB	0.05 ± 0.02aB	0.61 ± 0.08aA	0.4 ± 0.02bA	0.63 ± 0.03aA
Hypoxanthine	0.06 ± 0.01bB	0.09 ± 0.02aC	0.08 ± 0.01abA	0.07 ± 0.02bB	0.05 ± 0.01bB	0.12 ± 0.03aA	0.49 ± 0.08aA	0.15 ± 0.03bA	0.21 ± 0.12bA
L-Lysine	0.06 ± 0.02aB	0.09 ± 0.01aC	0.08 ± 0.04aB	0.04 ± 0.01aB	0.04 ± 0.01aB	0.07 ± 0.05aB	0.27 ± 0.03aA	0.31 ± 0.05aA	0.32 ± 0.03aA
Nicotinic acid mononucleotide	0.02 ± 0.01aA	0.02 ± 0.01aA	0.02 ± 0.01aA	0.10 ± 0.01aB	0.08 ± 0.01abB	0.06 ± 0.01bB	0.05 ± 0.01aB	0.04 ± 0.01bB	0.01 ± 0.01cB
Leucyl-isoleucine	0.04 ± 0.01aA	0.07 ± 0.01aA	0.05 ± 0.01aA	0.02 ± 0.02aA	0.03 ± 0.03aB	0.02 ± 0.02aB	0.01 ± 0.01bA	0.04 ± 0.01aAB	0.04 ± 0.01aAB
Aminoadipic acid	0.02 ± 0.01abA	0.02 ± 0.01aA	0.05 ± 0.01bA	0.15 ± 0.05aB	0.18 ± 0.04aC	0.12 ± 0.04aB	0.12 ± 0.01aA	0.12 ± 0.02aB	0.11 ± 0.01aA
1-Butylamine	0.01 ± 0.01aA	0.01 ± 0.01aA	0.05 ± 0.01aA	0.03 ± 0.01aB	0.03 ± 0.01aB	0.03 ± 0.01aB	0.01 ± 0.01aB	0.02 ± 0.01aAB	0.01 ± 0.01aB

Values are the means ± standard errors. Different lower-case letters indicate significant differences between strains under the same culture condition at *p* < 0.05. Different upper-case letters indicate significant differences in the same strain under different culture conditions at *p* < 0.05.

**Table 3:** Analysis of the relative content of commonly shared metabolites among three *E. sinensis* strains based on liquid chromatograph-mass spectrometer (LC-MS) negative ion (NEG).

Metabolites	Relative content of mycelium in PDB (%)			Relative content of fermentation broth in PDB (%)			Relative content of mycelium in PDA (%)		
	1	2	84C	1	2	84C	1	2	84C
Oleic acid	2.91 ± 0.14aA	2.65 ± 0.47bA	3.11 ± 0.35abA	0.01 ± 0.01aC	0.02 ± 0.01aC	0.02 ± 0.01aC	1.78 ± 0.29aB	1.24 ± 0.23bB	0.91 ± 0.22bB
Ketoleucine	1.67 ± 0.39aB	2.35 ± 0.31aB	2.36 ± 0.76aA	3.77 ± 0.83aA	3.43 ± 0.18aA	2.00 ± 0.65bA	0.36 ± 0.10aC	0.42 ± 0.18aC	0.29 ± 0.14aB
Myo-Inositol	1.39 ± 0.21aB	2.02 ± 0.16aB	1.67 ± 0.22aB	3.29 ± 0.59aA	3.32 ± 0.36aA	3.77 ± 0.50aA	0.11 ± 0.01aC	0.09 ± 0.01aC	0.07 ± 0.01bC
Uridine	0.92 ± 0.49aA	1.26 ± 0.55aA	0.82 ± 0.20aB	0.01 ± 0.01aB	0.01 ± 0.01aB	0.01 ± 0.01aC	1.33 ± 0.13aA	1.12 ± 0.16aA	1.37 ± 0.15aA
Uracil	0.75 ± 0.29aA	0.78 ± 0.16aA	0.99 ± 0.30aA	0.51 ± 0.07aB	0.53 ± 0.13aA	0.67 ± 0.12aAB	0.68 ± 0.07abAB	0.71 ± 0.09aA	0.53 ± 0.12bB
Pyrrolidonecarboxylic acid	0.64 ± 0.71abA	1.62 ± 0.95aB	0.13 ± 0.03bA	1.23 ± 1.17bA	3.07 ± 0.57aA	0.07 ± 0.01bC	0.14 ± 0.01aA	0.13 ± 0.01aC	0.10 ± 0.01bB
Succinic acid	0.51 ± 0.04bB	0.77 ± 0.02aB	0.64 ± 0.07bB	1.21 ± 0.20aA	1.31 ± 0.21aA	1.16 ± 0.17aA	0.39 ± 0.03bC	0.60 ± 0.13aB	0.54 ± 0.06abB
Phenyllactic acid	0.27 ± 0.12aB	0.43 ± 0.21aB	0.28 ± 0.04aB	1.11 ± 0.08abA	1.29 ± 0.23aA	0.88 ± 0.17bA	0.06 ± 0.01aC	0.06 ± 0.01aC	0.04 ± 0.01bC
D-Glutamine	0.20 ± 0.15aB	0.24 ± 0.11aB	0.28 ± 0.04aB	0.02 ± 0.01aC	0.01 ± 0.01aC	0.01 ± 0.01aC	0.48 ± 0.04bA	0.37 ± 0.04bA	0.79 ± 0.13aA
Quinic acid	0.11 ± 0.05abB	0.17 ± 0.02aA	0.09 ± 0.02bB	0.19 ± 0.12aAB	0.28 ± 0.11aA	0.19 ± 0.03aA	0.3 ± 0.04aA	0.16 ± 0.02cA	0.22 ± 0.02bA
Sorbitol	0.09 ± 0.03abB	0.14 ± 0.03aB	0.08 ± 0.02bB	0.09 ± 0.01aB	0.11 ± 0.02aB	0.09 ± 0.01aB	9.91 ± 0.51aA	8.65 ± 1.4abA	7.71 ± 0.37bA
Leucinic acid	0.06 ± 0.07aB	0.03 ± 0.02aB	0.10 ± 0.10aB	0.42 ± 0.06aA	0.33 ± 0.06abA	0.28 ± 0.04bA	0.05 ± 0.01aB	0.02 ± 0.01bB	0.02 ± 0.01bB
Aldehydo-D-xylose	0.06 ± 0.01aB	0.10 ± 0.06aB	0.10 ± 0.06aB	0.43 ± 0.08aA	0.41 ± 0.07aA	0.37 ± 0.02aA	0.06 ± 0.03aB	0.08 ± 0.03aB	0.07 ± 0.01aB
Formylanthranilic acid	0.05 ± 0.02aB	0.03 ± 0.01bC	0.05 ± 0.01abC	0.28 ± 0.07aA	0.13 ± 0.03bA	0.27 ± 0.03aA	0.08 ± 0.02aB	0.08 ± 0.03aB	0.12 ± 0.02aB
L-Serine	0.03 ± 0.01aB	0.04 ± 0.01aB	0.05 ± 0.01abB	0.01 ± 0.01aC	0.01 ± 0.01aC	0.01 ± 0.01aC	0.13 ± 0.01aA	0.12 ± 0.01aA	0.09 ± 0.01bA
5-Hydroxymethyluracil	0.03 ± 0.01abC	0.02 ± 0.01bB	0.05 ± 0.01aB	0.09 ± 0.02bB	0.06 ± 0.03bB	0.17 ± 0.02aB	0.72 ± 0.04bA	0.40 ± 0.03cA	0.93 ± 0.12aA
1,2,3-Trihydroxybenzene	0.02 ± 0.01abB	0.04 ± 0.01aB	0.02 ± 0.01bB	0.12 ± 0.05aA	0.13 ± 0.03aA	0.10 ± 0.02aA	0.04 ± 0.01abB	0.03 ± 0.01bB	0.04 ± 0.01aB
Maleic acid	0.01 ± 0.01aC	0.02 ± 0.01aC	0.02 ± 0.01aC	0.31 ± 0.03aA	0.27 ± 0.07aA	0.38 ± 0.08aA	0.12 ± 0.01bB	0.15 ± 0.02abB	0.18 ± 0.02aB
Threonic acid	0.01 ± 0.01aC	0.03 ± 0.02aC	0.02 ± 0.01aC	0.29 ± 0.02bB	0.26 ± 0.02bB	0.50 ± 0.06aB	0.63 ± 0.05aA	0.64 ± 0.03aA	0.65 ± 0.04aA
L-Iditol	0.01 ± 0.01aB	0.01 ± 0.01aB	0.01 ± 0.01aB	0.04 ± 0.02aB	0.02 ± 0.01aB	0.02 ± 0.01aB	0.27 ± 0.03bA	0.24 ± 0.04bA	0.56 ± 0.05aA
L-Gulose	0.01 ± 0.01bB	0.02 ± 0.01aB	0.01 ± 0.01aB	0.23 ± 0.05aA	0.17 ± 0.03aA	0.16 ± 0.03aA	0.03 ± 0.01aB	0.02 ± 0.01abB	0.01 ± 0.01bB

Values are the means ± standard errors. Different lower-case letters indicate significant differences between strains under the same culture condition at *p* < 0.05. Different upper-case letters indicate significant differences in the same strain under different culture conditions at *p* < 0.05.

## 4 Discussion

Overall, different strains of *E. sinensis* had similar metabolic pathways in the present study. The types, quantity and concentrations of secondary metabolites varied in different conditions (Oppong-Danquah et al., 2020). Our team preliminary research found that strain 1 (isolated from *F. sinensis* ecotypes in Xiahe, Gansu) exhibited a moderate growth rate, strain 2 (from *F. sinensis* ecotypes in Yushu, Qinghai) showed a faster growth rate, and strain 84C (from *F. sinensis* ecotypes in Ping’an, Qinghai) displayed a slower growth rate. Although these fungal endophyte strains from distinct geographical populations exhibit diversity in growth rate, colony morphology, and conidial characteristics, molecular identification confirms all belong to *E. sinensis* (Xu, 2022). In this study, differences were observed in the types of metabolites produced by the strains under PDA and PDB culture conditions. A total of 586 compounds were detected in the mycelium cultured in PDB, whereas 554 compounds were detected in the mycelium cultured in PDA. Additionally, a total of 635 compounds were identified in the fermentation broth from the PDB. However, varying incubation conditions lead to the production of different metabolites, and the number of compounds, organic heterocyclic and organic oxygen compounds in particular, in the PDB fermentation broth exceeded that found in the mycelial samples. The mycelium diameter, biomass and hormones (indoleacetic acid, cytokinin, gibberellin and abscisic acid) of the fungal endophytes were found to be significantly different between *E. sinensis* strains 1 and 84C under PDA and PDB (Luo and Tian, 2021). Liquid cultures provide larger mycelial yields in a shorter period of time, as well as allow for separation of mycelia and media, thus facilitating analysis of bioactivity of the secretome and intracellular metabolome separately (Kaur et al., 2015; Ekanayake et al., 2017). These differences confirmed that PDB medium has the advantage of uniform distribution of nutrients during microbial cultivation, which is conducive to full contact and absorption of nutrients by mycelia cells, and eventually increases the production of mycelia and nutrients (Luo and Tian, 2021). These findings confirm that the culture conditions have an important impact on the metabolism of the fungal endophytes.

Carbohydrates play dual roles as signaling molecules and stress protectants in plant-fungal systems (Sitepu et al., 2024). In this study, the relatively high-content commonly shared compounds include malic acid, adenosine monophosphate (AMP), proline, acetylcarnitine, oleic acid, ketoleucine, and inositol, with contents exceeding 1%. Other commonly shared compounds such as mannose, sorbitol, succinic acid, trehalose, uridine, uracil, and tyrosine have contents around 1%, while the contents of all other compound categories are extremely low. Mannose has been shown to be crucial to the glycosylation of some proteins (Brud and Koomey, 2024). Sorbitol has a chelating effect, combining with metal ions to form chelates, thereby maintaining internal moisture, preventing metal ions from combining with enzyme activity, and reducing protease activity (Lou and Chen, 2023). Malic acid and succinic acid serve as intermediates in the tricarboxylic acid (TCA) cycle, facilitating the synthesis of chemicals essential for mitigating oxidative stress through the regulation of respiration (Zhang et al., 2024). The accumulation of its content indicates that *E. sinensis* promotes the TCA cycle. Proline, an amino acid directly linked to the TCA cycle, possesses the ability to stabilize and protect biomacromolecules. It can be oxidized and coupled with the respiratory electron transport chain to generate reactive oxygen species (ROS) (Zhang and Becker, 2015). A reduced proline content may maintain structural integrity and function under drought stress (Tang et al., 2022). Adenine is converted into AMP in plant tissues to increase the concentration of ATP and plant growth (Merrett and Handoll, 2010). Trehalose, a stress-induced metabolite, forms a protective cell-surface film under extreme conditions (e.g., high temperature, hyperosmosis, and dehydration), thereby preserving molecular integrity and sustaining vital biological processes (Kim et al., 2024). Threitol may contribute to plant stress tolerance (Zhang et al., 2016). Our team has already researched the effects of *E. sinensis* on the interaction of host grasses *F. sinensis* with two fungal pathogens *Alternaria alternata* and *Bipolaris sorokiniana* were evaluated. *E. sinensis* endophyte improved disease resistance of the intact *F. sinensis* plants and host genotype also had effects on disease resistance (Wang Y. B. et al., 2021). However, *in vitro* culture conditions for fungal endophytes research result in a relatively low production of secondary metabolites. For some compounds with important biological functions, it is necessary to enrich mycelium under certain culture conditions and extract and separate single compounds to verify their functions. For example, a large number of crude extracts were derived from E + *A. inebrians* by ultrasonic combined with ethanol thermal reflux method, and pure ergot alkaloids were then separated from the crude extracts (Zhang et al., 2014). However, these compounds isolated from fungal endophytes grown *in vitro* can only be obtained by a large-scale hyphal collection under specific culture conditions.

In this study, KEGG annotation results on different metabolites showed that different compounds were mainly enriched in metabolic pathways, citrate cycle (TCA cycle) and alanine, aspartate, and glutamate metabolism. There were similar regulatory trends of metabolites for all comparison groups. Twenty metabolites were all downregulated in 84C vs. 1 and 84C vs. 2 groups, while 20 were upregulated in 1 vs. 2. The differential compounds in PDB broth between strains 1 and 2 had a broader range of enriched pathways, which largely overlapped with those found in PDA-harvested mycelia. In particular, the metabolic pathways with many different compounds being mapped were probably crucial for fungal endophytes to complete its metabolic functions *in vitro*. The metabolic pathways influenced by different compounds were more on PDA medium, indicating that the impact of PDA culture on the metabolic pathways was greater than that of the PDB culture.

## 5 Conclusion

In conclusion, the present study illlustrated that the new species *E. sinensis* produced diverse metabolites which varied with cultural conditions. The different strains shared the similar types of metabolites which only varied in concentrations.

## Data Availability

The raw metabolomics data has been uploaded to MetaboLights, accession number MTBLS13598 (https://www.ebi.ac.uk/metabolights/MTBLS13598).

## Footnotes

1
https://www.kegg.jp/kegg/pathway.html

2
https://cloud.metware.cn
